# Complete Genome Sequence of an F8-Like Lytic Myovirus (φSPM-1) That Infects Metallo-β-Lactamase-Producing *Pseudomonas aeruginosa*

**DOI:** 10.1128/genomeA.00061-14

**Published:** 2014-04-03

**Authors:** Patrícia R. Neves, Louise T. Cerdeira, Miguel Mitne-Neto, Théo G. M. Oliveira, John A. McCulloch, Jorge L. M. Sampaio, Elsa M. Mamizuka, Carlos E. Levy, Maria I. Z. Sato, Nilton Lincopan

**Affiliations:** a Department of Microbiology, Institute of Biomedical Sciences, Universidade de São Paulo, São Paulo, Brazil; b Research and Development Department, Fleury Group, São Paulo, Brazil; c Laboratory of Genetics and Molecular Cardiology, Heart Institute (InCor), School of Medicine, Universidade de São Paulo, São Paulo, Brazil; d Department of Clinical Analysis, School of Pharmacy, Universidade de São Paulo, São Paulo, Brazil; e Department of Clinical Pathology, School of Medicine, Universidade de Campinas, Campinas, Brazil; f Environmental Company of São Paulo State (CETESB), São Paulo, São Paulo, Brazil

## Abstract

*Pseudomonas aeruginosa* is an important cause of infection, especially in immunocompromised patients. In this regard, strains producing carbapenemases, mainly metallo-β-lactamases (MBLs), have become a significant public health concern. Here, we present the complete annotated genome sequence (65.7 kb) of an F8-related lytic myovirus (*Pbunalikevirus* genus) that infects MBL-producing *P. aeruginosa* strains.

## GENOME ANNOUNCEMENT

Metallo-β-lactamase (MBL)-producing *Pseudomonas aeruginosa* strains have become a significant public health problem (1). Here, we report the sequencing and annotation of a lytic myovirus infecting MBL-producing *P. aeruginosa*.

A lytic myovirus, called φSPM-1, was recovered from a collection of environmental isolates (from surface water samples from rivers in southeastern Brazil) and oldest-typing-set phages (2); it was propagated in an environmental SPM-1-producing *P. aeruginosa* host strain (3) and then was characterized by transmission electron microscopy and complete-genome sequencing. This phage was able to lyse clonally unrelated MBL-producing *P. aeruginosa* strains (4), including the Brazilian endemic clone harboring the *bla*_SPM-1_ MBL gene, sequence type 277 (ST277) (5). The phage had an elongated icosahedral head with an approximately 50-nm diameter and a tail near 70 nm in length, showing that it belongs to the *Myoviridae* family of the order *Caudovirales* (6).

The bacteriophage genome was sequenced by Ion Torrent PGM (Life Technologies) using the Ion Xpress Plus fragment library kit, with size selection by electrophoresis before emulsion PCR on an Ion OneTouch apparatus, using the Ion PGM sequencing 400 and Ion PGM template OT2 400 kits. A total of 82,540 reads (~200× coverage), with a mean length of 159 bp, were obtained after sequencing, and *de novo* assembly of next-generation sequencing (NGS) reads (CLC bio Genomics Workbench version 6.5) yielded 19 contigs (7).

The whole-genome sequence (WGS) of the myophage was assembled using crossmatch alignment (40-bp minimum overlap), and the prediction of protein-coding sequences (CDSs) was obtained using phage annotation tools and methods (PhAnToMe) (http://www.phantome.org). Manual curation of opening reading frames (ORFs) was carried out by comparative genomics using BLASTp (http://www.ncbi.nlm.nih.gov/blast/Blast.cgi) analysis of proteins against the GenBank NR database.

The WGS of φSPM-1 comprises 65,729 bp, with a G+C content of 54.97%, 92 ORFs, and no tRNAs, harboring genetic information needed for replication of nucleic acid and synthesis of protein coats (i.e., genes coding for DNA replication, capsid, tail, packaging, and lytic enzymes). Moreover, the φSPM-1 genome is 95.6% identical to that of the *P. aeruginosa* F8 phage (GenBank accession no. NC_007810.1). In this regard, a 237-bp region, which is absent in φSPM-1 and present in the F8 phage, partially encodes a hypothetical protein of unknown function in the F8 phage genome. The start point of the F8 genome sequence was positioned at a locus that possesses a CDS that is predicted to code for a tail-length tape-measure protein (locus tag PPGF8SP_0065). We have therefore marked the genome sequence as circular. In this respect, the linear supercontig was extended by adding 100 ambiguous “N” bases on either side. Alignment of the reads to this supercontig was then carried out using CLC Genomics Workbench, and their extension was possible in regions that had a read coverage of >10× and that spanned the supercontig ends and the ambiguous “N” bases added; this yielded a 40-bp overlap of the extension, circularizing the sequence.

Finally, the φSPM-1 phage genome has high identities to other *P. aeruginosa* phages belonging to the *Pbunalikevirus* genus (8) (i.e., PB1 [93.3%], LBL3 [89.9%], LMA2 [87%], 14-1 [86.9%], and SN [87%] phages), as analyzed using Geneious software (Biomatters Ltd.).

### Nucleotide sequence accession number.

The complete genome sequence of F8-like lytic myovirus φSPM-1 (*Pbunalikevirus* genus) was deposited in GenBank with the accession no. KF981875.
